# Nuclear Receptors and Endocrine Disruptors in Fetal and Neonatal Testes: A Gapped Landscape

**DOI:** 10.3389/fendo.2015.00058

**Published:** 2015-05-07

**Authors:** Virginie Rouiller-Fabre, Marie Justine Guerquin, Thierry N’Tumba-Byn, Vincent Muczynski, Delphine Moison, Sophie Tourpin, Sébastien Messiaen, René Habert, Gabriel Livera

**Affiliations:** ^1^Unit of Genetic Stability, Stem Cells and Radiation, Laboratory of Development of the Gonads, Sorbonne Paris Cité, Université Paris Diderot, Fontenay-aux-Roses, France; ^2^CEA, DSV, iRCM, SCSR, LDG, Fontenay-aux-Roses, France; ^3^Unité 967, INSERM, Fontenay aux Roses, France

**Keywords:** DES, BPA, phthalates, nuclear receptors, human fetal testis

## Abstract

During the last decades, many studies reported that male reproductive disorders are increasing among humans. It is currently acknowledged that these abnormalities can result from fetal exposure to environmental chemicals that are progressively becoming more concentrated and widespread in our environment. Among the chemicals present in the environment (air, water, food, and many consumer products), several can act as endocrine disrupting compounds (EDCs), thus interfering with the endocrine system. Phthalates, bisphenol A (BPA), and diethylstilbestrol (DES) have been largely incriminated, particularly during the fetal and neonatal period, due to their estrogenic and/or anti-androgenic properties. Indeed, many epidemiological and experimental studies have highlighted their deleterious impact on fetal and neonatal testis development. As EDCs can affect many different genomic and non-genomic pathways, the mechanisms underlying the adverse effects of EDC exposure are difficult to elucidate. Using literature data and results from our laboratory, in the present review, we discuss the role of classical nuclear receptors (genomic pathway) in the fetal and neonatal testis response to EDC exposure, particularly to phthalates, BPA, and DES. Among the nuclear receptors, we focused on some of the most likely candidates, such as peroxisome-proliferator activated receptor (PPAR), androgen receptor (AR), estrogen receptors (ERα and β), liver X receptors (LXR), and small heterodimer partner (SHP). First, we describe the expression and potential functions (based on data from studies using receptor agonists and mouse knockout models) of these nuclear receptors in the developing testis. Then, for each EDC studied, we summarize the main evidences indicating that the reprotoxic effect of each EDC under study is mediated through a specific nuclear receptor(s). We also point-out the involvement of other receptors and nuclear receptor-independent pathways.

## Introduction

### Endocrine disruptors and male reproductive function

During the last 50 years, the frequency of various male reproductive disorders, such as cryptorchidism, hypospadias, testicular cancer, and low sperm count, has progressively increased. Many clinical, epidemiological, and experimental studies suggest that these disorders arise during fetal development (1, 2).

The fetal period is critical for proper testis development. Indeed, gametogenesis and steroidogenesis, the two major functions of the testis, are set up during this period and their proper onset is fundamental for the adult reproductive function. For instance, the number of germ cells formed during fetal life will strongly affect adult fertility. In mutant mice with pronounced germ cell loss during fetal life, adult fertility is altered (3, 4). Similarly, exposure of mouse perinatal testes to gamma rays decreases the sperm counts at sexual maturity (5). Concerning steroidogenesis, fetal Leydig cells produce testosterone that is responsible for the masculinization of the male genital tract and external genitalia (6, 7). Importantly, androgens must act during a specific period, called the «masculinization programing window»that predates genital masculinization. Genital development in the rat is programed between 15.5 and 18.5 day post-coitum (dpc). This corresponds to the 13.5–17.5 dpc period in the mouse and to the 6.5–14 gestational week (GW) time in humans (8). Fetal androgens are also required for the proper development of adult Leydig stem cells (9) and for testis descent, which depends also on Insulin-like 3 (INSL3), another hormone produced by fetal Leydig cells (10, 11).

It has been hypothesized that male reproductive system abnormalities could be related to the massively increased presence in the environment of natural and synthetic chemicals during the last decades (1, 12, 13). Among the chemicals present in air, water, food, and in a variety of consumer products, many can interfere with the endocrine system and therefore are called endocrine disrupting compounds (EDCs). EDCs can affect the production, release, transport, metabolism, binding or elimination of natural hormones (2). Among the EDCs present in the environment, phthalates, bisphenol A (BPA), and diethylstilbestrol (DES) have been chiefly incriminated, particularly during the fetal and neonatal period due to their estrogenic and/or anti-androgenic properties (14–17).

A large amount of data shows the effects of EDCs on testis development. EDCs can act by affecting different tissue-specific genomic and non-genomic pathways. It is now crucial to identify which nuclear receptors and downstream signaling pathways are altered by exposure to DES, BPA, or phthalates in human and rodent fetal gonads.

In the present study, we highlight the involvement of some classical nuclear receptors in the response of fetal and neonatal testes to DES, phthalates, and BPA exposure. We focused particularly on peroxisome-proliferator activated receptor (PPAR), androgen receptor (AR), estrogen receptors (ER1 and 2), liver X receptor (LXR), and small heterodimer partner (SHP) due to their affinity or involvement in mediating some EDC effects (Table 1).

**Table 1:** **Nomenclature and classification of the chosen receptors**.

Families	Group	Member	Acronym	Name
NR1	C	NR1C1	PPARα	Peroxisome-proliferator-activated receptor alpha
		NR1C2	PPARβ/δ	Peroxisome-proliferator-activated receptor beta
		NR1C3	PPARγ	Peroxisome-proliferator-activated receptor gamma
	H	NR1H2	LXRβ	Liver X receptor beta
		NR1H3	LXRα	Liver X receptor alpha
NR3	A	NR3A1	ERα	Estrogen receptor alpha
		NR3A2	ERβ	Estrogen receptor beta
	C	NR3C4	AR	Androgen receptor
NRO	B	NR0B2	SHP	Small heterodimer partner

*From Giguere V, Endocrine review 1999, and the Nuclear Receptor Nomenclature Committee*.

## Expression and Roles of Nuclear Receptors in Fetal and Neonatal Testes

**Figure 1:**
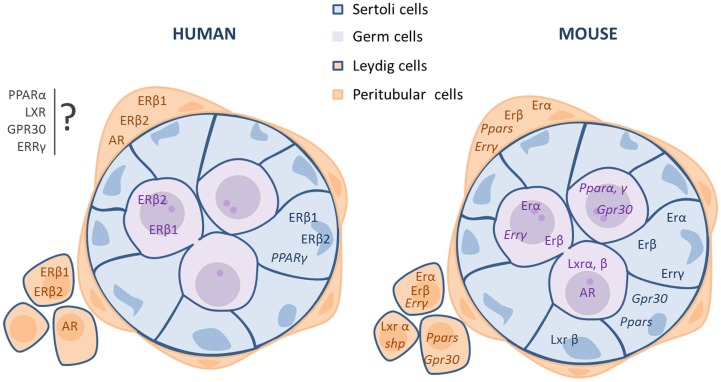
**Localization of the receptors involved in EDCs effects in fetal or neonatal mammalian testes (human and rodents)**. The mRNA (*italic type*) or protein (roman type) expression of the receptors is indicated in each cell type: germ, Sertoli, and interstitial (Leydig and peritubular cells) cells. *Potential localization in somatic cells.

Here, we describe the expression profile of these nuclear receptors in the male gonad based on literature and personal data (Figure 1).

### Estrogen receptors

Estrogen action is mainly mediated through the activation of two specific estrogen receptors (ER), ERα (ESR1/NR3A1) and ERβ (ESR2/NR3A2), in target cells (18–21). The expression of these two receptors in the testis varies according to the species and developmental stage (Figure 1).

In rat fetal testes, ERβ protein is present in all cell types, whereas ERα is expressed only in Leydig cells (22). In the mouse, immunohistochemical studies showed that ERα is expressed in undifferentiated gonads as early as 10.5 dpc and then is localized in fetal Leydig cells until birth, while it is absent in germ cells during the fetal period (23, 24). ERα signaling is functional in the fetal testis, as ERα knockout leads to an increase in testosterone production by mouse fetal testes as soon as 13.5 dpc (25). ERβ is expressed in rat and mouse testis germ cells (Figure 1) from 14.5 dpc (26). In the rat, it is also expressed in Sertoli and Leydig cells from 16.5 dpc (26–28). In sorted cells from mouse fetal testes, we detected that ERα mRNA level is higher than that of ERß. Interestingly, ERα transcripts are mainly detected in germ cells, but also in interstitial and Sertoli cells (Figure 1). ERβ is mainly expressed in interstitial cells and to a lower extent in Sertoli and germ cells (Figure 1).

In humans, ER α and β expression in the testis have been assessed mainly during the second quarter of pregnancy (Figure 1). These studies highlighted the presence of two ERß variants (ERß1 and ERß2) (29, 30) that are expressed (mRNA) in Sertoli and germ cells and also in some interstitial cells (29, 31, 32). Conversely, ERβ1 protein is specifically detected in Sertoli and interstitial cells, whereas ERβ2 protein is only detected in germ cells (32). Concerning ERα expression, some studies did not detect neither mRNA nor protein expression in human fetal testis (31–33), while others detected both (29, 34).

Estrogen effects in the testis have been elucidated using genetically modified mice that lack ERα (ERαKO), ERβ (ERβKO) (35), or both (ERαβKO), or the CYP19/aromatase gene (ArKO) (36, 37). ERαKO, ERβKO, ERαβKO, and ArKO male mice show reproductive disorders. ERαKO and ArKO mice are sterile due to epididymal fluid reabsorption deficiency and spermatogenesis disorders, respectively (14, 38). Endogenous estrogens also play an important role during fetal and neonatal testis development (25, 39). ERβ knock out induces a 50% increase in the number of gonocytes observed after birth, due to increased proliferation and decreased apoptosis (39), but no changes during fetal life. Conversely, ERα gene deletion does not modify the number of gonocytes, but increases testosterone production during fetal life from 13.5 dpc (25). Thus, ERβ is involved in the control of neonatal gametogenesis, whereas ERα regulates fetal and neonatal steroidogenesis.

Estrogens play a vital role in the control of human reproductive functions as well. Studies in patients with *ER*α or *CYP*19 inactivating mutations suggest a role of estrogens in human male fertility (40).

### Androgen receptors

Androgen actions are mediated through the AR (Figure 1). Androgen and AR roles in the masculinization of male genitals are well known. AR mutations cause androgen insensitivity syndrome (AIS), possibly the most frequently described hormone insensitivity syndrome. The most serious AIS phenotype is testicular feminization (Tfm) where genetic males are females in appearance. In adult life, androgen action on seminiferous tubules is essential for normal spermatogenesis and fertility and most evidences suggest that this effect is mediated by Sertoli cells (41). In adult testis, AR is expressed in Sertoli cells in almost all species tested, but also in peritubular cells, Leydig cells, and spermatids (42).

In the mouse, AR mRNA and protein are expressed in germ cells during fetal life (43), when gonocytes are the main testis cell type physiologically controlled by endogenous androgens. Leydig cells are largely independent of endogenous androgens during fetal development. On the other hand, peritubular myoid and Sertoli cells seem to become androgen-dependent mainly in the latest part of fetal development (44). However, during late fetal life, androgen positive effect on Sertoli cell proliferation is probably indirect because AR is expressed in Sertoli cells only after birth (45). In human fetal testis, AR is expressed in peritubular and Leydig cells, but not in germ or Sertoli cells (46) (Figure 1).

Few studies exist on androgen and AR functions in fetal testis. Nevertheless, alterations of testis functions resulting from exposure to anti-androgenic EDCs during fetal testis development suggest a key role for androgens and AR during this period. AR knock out (and thus decreased fetal androgen signaling) in mice leads to a reduction of intratesticular testosterone level and of the number of adult Leydig stem cell by 40% at birth to adulthood (9). Similarly, the analysis of a mouse model in which AR was selectively invalidated in Leydig cells from fetal life onward and of patients with complete AIS showed that androgen autocrine action is essential for Leydig cell maturation and function (47). Finally, increased prenatal exposure to androgens alters the development and function of Leydig cells at a time when androgen production is paramount for male development (48). Similarly, in male lambs exposed prenatally to an excess of testosterone, the number of Sertoli cells is increased and this effect could explain the testicular dysfunction observed in adult rams (49).

Concerning androgen role in germ cells, AR deficiency results in increased gonocyte numbers during fetal life due to higher proliferative activity in Tfm mice. Conversely, gonocyte proliferation is decreased by the addition of DES in fetal testis organotypic cultures (43).

### Peroxisome-proliferator activated receptors

The PPAR family includes three members: PPARα (NR1C1), PPARβ/δ (NR1C2), and PPARγ (NR1C3). They are mostly involved in lipid and cholesterol metabolism and in fatty acid synthesis (50). They are also present in the female and male reproductive organs (51).

In adult rat testis, the three PPAR members are located in the interstitial space and in the seminiferous cords (52) (Figure 1). PPARβ/δ is the most expressed isoform in the testis, followed by PPARα. Although PPARγ protein is barely detectable (52), PPARγ mRNA expression was detected mostly in Sertoli cells (53), but also in spermatocytes (54) and in fetal germ cells (55). In mouse fetal testes, PPARα is mainly expressed in interstitial cells, but also in Sertoli and germ cells. PPARγ is mostly expressed in Sertoli cells and then in interstitial and germ cells (personal data Figure 1). PPARγ expression is higher in testicular cancer cells than in normal cells (56).

For human fetal testes, we developed a novel flow cytometric cell sorting approach based on the staining of M2A, a trans-membrane antigen that is expressed in 90–95% of germ cells during the first trimester of pregnancy. The M2A-positive cell fraction expresses only germ cell markers, whereas the M2A-negative fraction expresses exclusively somatic cell markers. We have shown that PPARγ mRNA is expressed only in M2A-positive cells (55) (Figure 1).

Concerning PPAR implication in the reproductive function, PPARα deletion in mice did not have any effect on their viability and fertility (57). Furthermore, gestational exposure to a PPARα agonist did not reduce fetal testicular testosterone production (58). However, PPARα and PPARβ/δ have been recently identified as regulators of Sertoli cell metabolism (59). As PPARγ knockout mice die at 10 dpc (60), its effect on the reproductive functions could not be tested. The results of microarray analyses suggest that PPARγ could play an important role in regulating the expression of key lipid metabolic genes in Sertoli cells during postnatal development (61).

### Liver X receptors

The liver X receptors LXRα (NR1H3) and LXRβ (NR1H2) belong to a subclass of nuclear receptors that form obligate heterodimers with retinoid X receptors (RXRs) and that are activated upon binding of their ligands (oxysterols) (62). They are mainly involved in the regulation of cholesterol and fatty acid homeostasis, but also in glucose homeostasis, immunity, skin development, and brain functions (63).

In the mouse, quantitative PCR analysis has shown that LXRα is expressed in Leydig cells, LXRβ in Sertoli cells, and both in germ cells (64) (Figure 1). In human fetal testes, we demonstrated, using our cell sorting approach, that LXRα mRNA is expressed both in M2A-negative and in M2A-positive cells (55) (Figure 1).

Recently, their physiological role in the regulation of male reproductive functions has been elucidated using LXRα and LXRβ single and double knockout mouse models (64). Mice in which LXRα or LRXβ has been invalidated show reduced fertility throughout life, whereas double knockout mice show reduced fertility at 5 months which progresses to complete sterility by the age of 9 months (65). Moreover, LXRs seem to have distinct roles in sustaining spermatogenesis (66, 67). Indeed, germ cell apoptosis is increased in 3.5-month-old LXRα-deficient mice, whereas the number of proliferating germ cells is reduced in LXRβ-deficient mice compared to wild type animals. Therefore, deregulation of one of the LXR pathways can be easily related to the disruption of the fine equilibrium between germ cell proliferation and apoptosis, leading to alterations of the reproductive function. For instance, a recent study has reported a positive correlation between reduced number of germ cells and decreased LXR mRNA level in testis biopsies of patients with various degrees of azoospermia (68). Furthermore, in 2.5-month-old LRXα-deficient mice, testosterone production is significantly lower than in wild type controls and the level of 3β-hydroxysteroid dehydrogenase isomerase mRNA (an enzyme that plays a crucial role in the biosynthesis of hormonal steroids) is significantly reduced in these mice (65, 66).

### Small heterodimer partner

Small heterodimer partner (SHP/NR0B2) is a member of the nuclear receptor superfamily and is classified as an “orphan” receptor, because its ligand has not been identified yet. This receptor is mainly known for its role in liver and in the control of bile acid homeostasis (69, 70).

Small heterodimer partner expression in the testis is very low (62). In a study performed with purified cells (using laser microdissection), SHP mRNA was mostly expressed in interstitial cells of adult mouse testis. SHP is also transiently expressed in the tubular cells of seminiferous tubules during early postnatal development and its expression declines as the mice reach sexual maturity (71, 72) (Figure 1). Interestingly, SHP is not detectable in human fetal testes (55).

Small heterodimer partner deletion in mice results in higher testosterone production due to enhanced expression of steroidogenic genes, such as steroidogenic acute regulatory protein (StAR) and CYP11A1 (71). SHP knockout mice also show earlier differentiation of germ cells compared with control littermates, as suggested by the number of tubules with elongated spermatids.

The retinoic acid (RA) metabolic pathway is affected by SHP knock out as indicated by the altered expression of several RA receptor (RAR) target genes in these mice. Among them, Stra8, a key gene in meiotic initiation (73, 74), is up-regulated, possibly as a result of the increased RA expression in the testes of NR0B2^−/−^ mice (71).

## Endocrine Disruptors and Male Reproductive Functions: Involvement of Nuclear Receptors

### Diethylstilbestrol

#### Diethylstilbestrol and Male Reproductive Function

The so-called “estrogen hypothesis,” which was first proposed 20 years ago, suggests that the growing incidence of male reproductive abnormalities in humans could be related to increased estrogen exposure (75). Over the years, this hypothesis has been supported by a large number of epidemiological and experimental studies.

Diethylstilbestrol is a synthetic estrogen and a recognized EDC. Between 1945 and 1971, DES was administered to pregnant women to prevent miscarriage or premature delivery. This was associated with an increased incidence of reproductive tract abnormalities in their male and female offspring (75). Specifically, several studies have reported alterations in sperm quality and higher incidence of genital malformations, cryptorchidism, and testicular cancer than in untreated populations (76, 77). DES seems to have a negative effect on sperm count when administered at high dose during the first semester of pregnancy (78).

Similarly, experimental studies support the estrogen hypothesis (14), *in utero*, rodents exposed to DES during development have abnormal testicular histology and altered adult male fertility (14, 79). Also, *in utero* exposure to DES of 9 and 10 dpc rat embryos can advance testicular development, alter the differentiation of gonocytes and Sertoli cells, and cause fetal Leydig cell hyperplasia from 16 dpc onward (33, 80–82). In the same way, *in vitro* incubation of rat fetal testes with DES reduces testosterone production and the number of gonocytes by inhibiting proliferation and increasing apoptosis (83, 84). Likewise, in a mouse fetal testis organ culture system, DES exposure reduces testosterone production (25). *In utero* exposure to DES also alters mouse testis development by decreasing testosterone levels in testes and reducing the expression of StAR (85) (Table 2).

**Table 2:** **Involvement of nuclear receptors in EDCs effects in human and mouse testes**.

	Receptors potentially involved in EDC response	Testicular alterations			
		Leydig cells		Germ cells	
		Testosterone	Secretion	Number	Plurinuclei
DES	ERs, NROB2, LXRs	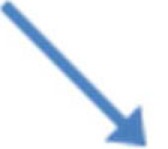	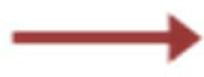	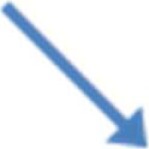 *nd*	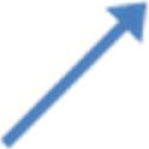 *nd*
Phthalates	PPARs, LXRs	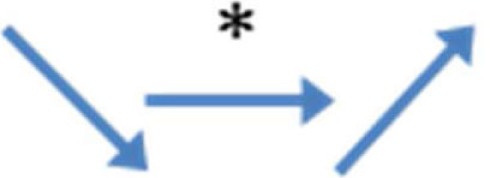	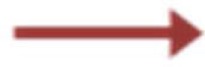	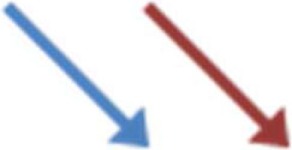	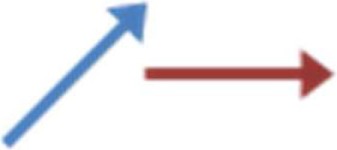
BPA	ERs	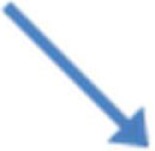	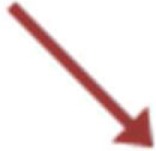	*nd*	*nd*

*Blue arrows: mouse, red arrows: human*.

*nd: not determined; ↓: “decrease”; ↑: “increase”; →: “no effect”*.

*In mouse, the effects of phthalates on steroidogenesis vary with the developmental stages and experimental conditions.

Surprisingly (relative to the epidemiological data), testosterone secretion by cultured human fetal testes is barely affected by DES exposure, while a strong effect is observed in mouse testes (33, 55) (Table 2).

#### DES and Nuclear Receptors

##### DES and ERs

Diethylstilbestrol exerts its anti-androgen effects mainly through classical ER signaling, particularly via ERα (25, 86). In an organ culture system of mouse fetal testes, the reduction in testosterone production observed following DES exposure in wild type testes does not occur in ERα-deficient mice (25). Similarly, INSL3 gene expression and testis descent are not affected by *in utero* exposure to DES in ERαKO mice, whereas ERβ invalidation does not protect from DES effect (86).

##### DES and ARs and PPARs

To our knowledge, there is no data showing the direct involvement of AR or PPARs in DES testis effects. However, an indirect action of DES cannot be excluded in relation with the reduction of testosterone secretion observed *in vitro* in rodent testes incubated with DES (14).

##### DES and LXRs

Some studies have linked estrogens and LXRs in breast and in mouse adipose tissue (87, 88). In the testis, LXRs could partially interfere with DES effects. Daily treatment with DES from day 1 to day 5 after birth induces an important increase in cell apoptosis in LXR-deficient mice at day 10 compared to wild type animals. Likewise, LXRs modify the neonatal effects of DES on the expression of Leydig and Sertoli cell markers (67). However, whether LXRs have a protective effect against or contribute to DES effects remains unclear.

##### DES and SHP

Treatment with DES promotes SHP mRNA accumulation in the testes of wild type SHP male mice (NR0B2^+/+^) at postnatal day 10 (P10). Moreover, neonatal DES exposure induces apoptosis, in P10 NR0B2^+/+^ mice, without any effect on cell proliferation. Conversely, DES does not have any effect on apoptosis in the testes of NR0B2^L−/L−^ males, suggesting that SHP inactivation protects against DES effects. This seems to be germ cell-specific because DES treatment drastically decreases intratesticular testosterone in both NR0B2^L−/L−^ and NR0B2^L−/L−^ males (89). Interestingly, SHP, mediating the deleterious effects of DES in mice, is not detectable in human fetal testes, and incubation with DES does not modify testosterone production by human fetal testes in culture (90). SHP absence in human fetal testes could be an additional explanation for their lack of sensitivity to DES.

### Phthalates

#### Phthalates and Male Reproductive Function

Phthalates (phthalic acid esters) are industrial chemicals commonly found in many consumer products, such as soaps, shampoos, cosmetics, and hairsprays. They are also used in flexible plastics, such as food and beverage packaging, children’s toys, and biomedical equipment (91). Phthalates are not covalently bound to plastic products and therefore may leak out. Di-2-ethylhexyl phthalate (DEHP) is the most produced phthalate. In the gut, liver, and blood, DEHP is rapidly hydrolyzed by esterases into its monoester metabolite mono-2-ethylhexyl phthalate (MEHP), considered to be the active metabolite and a recognized active testicular toxicant (55, 92–95), although DEHP, the parental compound, is also suspected to affect steroidogenesis (93).

Epidemiological studies in humans did not clearly prove the association between masculinization defects and phthalate exposure during fetal life (96, 97). Conversely, experimental studies in rats have shown that phthalates (di-butyl phthalate, DBP) administered during fetal life *in vivo* reduce testosterone production (98) (Table 2). This effect could be due to a decrease in Leydig stem/progenitor cells (9).

Surprisingly, MEHP does not affect *in vitro* testosterone production in human fetal testis explants (94). This was surprising because phthalates are considered to be anti-androgenic compounds based on their *in vivo* inhibitory action on testosterone production in the rat (7, 16, 99, 100). The recent observations that DBP decreases the steroidogenic activity of rat fetal testes, but not of human fetal testes, grafted into a mouse or rat host (101–103) definitively confirm that phthalates are not anti-androgenic compounds for human fetal testis (Table 2).

Phthalates also impair gonocyte development in the rat (104, 105) and increase gonocyte apoptosis *in vitro* in rat (93) and mouse fetal testes (95). Using an organotypic culture system, we demonstrated that MEHP also induces germ cell apoptosis in human fetal testes (94) after only 3 days of culture and at doses (10^−5^M) in the range of human exposure. *In vitro*, MEHP induces the appearance of multinucleated gonocytes (MNGs) in mouse and rat, but not in human fetal testis explants. However, exposure of human fetal testis xenografts to 100–500 mg/kg of DBP significant increases MNG content compared with untreated control (101).

#### Phthalates and Nuclear Receptors

##### Phthalates and PPARs

Peroxisome-proliferator activated receptor activation has been widely linked to the adverse effects of exposure to phthalates in adult mice (106). PPAR role in phthalate toxicity was first demonstrated in the liver (107).

Several studies using reporter genes for the different PPARs showed that phthalates can directly activate PPARs, particularly PPARα and PPARγ (53, 108).

*In vitro* transactivation assays demonstrated that MEHP can activate human and rodent PPARα and PPARγ (109). However, in PPARα knockout mice, the deleterious effects of DEHP exposure on male genitals were only partially blocked, suggesting that some of the toxicological effects of phthalate esters are mediated also by other nuclear receptors (57, 110).

A large risk assessment study demonstrated PPAR involvement in di-isobutyl phthalate (DiBP) toxic effects in the testis (111). Particularly, DEHP or DiBP treatment between 7 and 21 dpc induces an increase in PPARα gene expression and PPARγ protein expression in Leydig cells of fetal rat testes (112, 113). Moreover, DEHP induces apoptosis in adult rat testes via PPARγ and the ERK1/2 pathway (114). We demonstrated that MEHP (55, 94, 95) and PPARα or PPARγ agonists (unpublished data) have similar apoptotic effects in mouse and human fetal germ cells.

##### Phthalates and ERs

Phthalate actions in the developing testis seem independent of ER signaling. A study performed in our laboratory showed that the effects of phthalates on steroidogenesis vary with the developmental stage. Conversely, the strong deleterious effect of phthalates on germ cells is present during the active phases of gonocyte development and thus is unaffected by the steroidogenic status. Moreover, phthalate effects were comparable in testes from ERαKO or ERβKO mice and wild type controls (95). Interestingly, it has been suggested that the onset of dysgenesis in fetal rat testes exposed to phthalates may be mediated by estradiol (115).

##### Phthalates and ARs

Studies performed in our laboratory have shown that after MEHP treatment, (MNGs are similarly increased in testes from AR-deficient mice (Tfm) and wild type controls. Furthermore, incubation with MEHP for 3 days reduces the number of gonocytes by ∼40–50% in cultured 18.5 dpc testes from both wild type and Tfm mice. These results demonstrate that MEHP reduces the number of germ cells independently of the AR pathways (95).

##### Phthalates and LXRs

LXRs have also been related to the deleterious effect of exposure to environmental pollutants, particularly phthalates, during fetal life. Exposure of human fetal testis to MEHP during the first trimester of pregnancy leads to up-regulation of LXRα mRNA expression specifically in testis somatic cells, but not in germ cells (55). This effect is associated with increased transcription of Sterol Response Element Binding Protein 1c (SREBP1c) and downstream effectors involved in cholesterol and lipid synthesis and lipid accumulation in somatic cells. Somatic cells support the testis architecture that is essential for maintaining germ cell development. Therefore, the MEHP-induced modulation of LXRα expression specifically in these cells could lead to a deregulation of cell interactions, ultimately increasing germ cell apoptosis and reducing their number (94, 116).

##### Phthalates and SHP

To our knowledge, there is no data on SHP involvement in phthalate testis effects. However, LXRs can be modulated by SHP (89), thus suggesting a potential complex mechanism of EDC effect through several nuclear receptors.

### Bisphenol A

#### Bisphenol A and Male Reproductive Function

Bisphenol A [BPA, 2,2-Bis (4-hydroxyphenol) propane] is one of the most studied EDCs. BPA was first synthesized by Dianin in 1891 and its estrogenic activity was discovered in 1936 (117). It is, therefore, one of the oldest synthetic compounds known for its endocrine activity, although DES was preferred because of its stronger estrogenic activity.

Bisphenol A is widely used as a monomer for the industrial production by polymerization of polycarbonate plastics (72%) that are used in a variety of common products (optical, media, automotive, electrical and electronics, housewares and appliances, construction, medical, packaging…). BPA is also used (21%) as an essential component of epoxy resins that are mainly employed to coat the inner surface of food and beverage metallic cans (118). Lastly, BPA is used as anti-oxidant or inhibitor of polymerization in some plasticizers, polyvinyl chloride, and in thermal cash register paper (119).

BisphenolA can leach into the content of food containers made of polycarbonate plastic or coated with epoxy resins and then be ingested. This is the main source of human contamination, although its ubiquitous distribution leads also to contamination from dermal exposure through the skin, especially from thermal paper, and from inhalation of household dusts.

Growing evidence from research on laboratory animals, wildlife, and humans supports the view that BPA has an endocrine disrupting effect and adversely affects male reproductive function (100, 120).

In humans, most studies reported the association between masculinization defects and BPA exposure during fetal life. In China, sons of workers who were occupationally exposed to BPA during pregnancy showed shorter anogenital distance (AGD) (121). In the general Korean population, BPA level in the plasma of newborn boys with hypospadias was seven times higher than in newborns without hypospadias, and the difference was statistically significant (122). Lastly, although no BPA increase was first detected in newborn boys with undescended testes in France (123), recent data from this group showed a negative correlation between the cord blood concentrations of BPA and Insulin like 3 (INSL3) (124).

The *in vivo* effects of BPA on fetal Leydig cell function in rodents are not clear. Some studies reported an inhibitory effect of BPA on plasma testosterone at birth (125) or on the AGD in male pups (126), but others did not (127–130). Recently, using an *in vitro* organotypic culture system, we analyzed the testosterone production of mouse, rat, and human fetal testes in response to various doses of BPA. At high doses, BPA reduces testosterone production in all three species. However, only human fetal testes are sensitive to lower doses (10^−8^M). Furthermore, BPA treatment reduces *INSL3* mRNA level in human testes (by more than 20%), but not in rat and mouse testes (17, 90) (Table 2). This might explain the difficulty to observe a masculinization defect associated with BPA exposure in rodents (above).

Very few studies have investigated in detail BPA effects on fetal germ cells. Exposure *in utero* to low BPA concentrations by daily oral administration to pregnant female rats induces a decrease in litter size and in sperm number and motility in the adult progeny. It is important to note that these effects are maintained in the subsequent generations (F2, F3), suggesting a reprograming of germ cells by (genetic/epigenetic) mechanisms that persists well beyond the initial exposure (131). A potential explanation is that BPA exposure could affect DNA methylation of imprinted genes in fetal mouse germ cells (132).

#### BPA and Nuclear Receptors

##### BPA and ERs

Using *in vitro* bioassays based on competitive binding to nuclear receptors, reporter gene expression, and cell proliferation assessment, it has been shown that BPA efficiently activates both ERs (133).

Bisphenol A binds to ERα and ERß, but its affinity for these receptors is weak (∼1000-fold lower than estradiol) (134). This low affinity and the non-detectable expression of ERα in human fetal testes (32, 33, 135) suggest that ERα is not involved in BPA effect in humans. This is probably true also for the mouse species because the negative effect of BPA on mouse testis steroidogenesis is maintained after ERα invalidation (90).

##### BPA and ARs

In reporter cell lines, BPA or its halogenated derivatives inhibit ARs (136). Using *in vitro* bioassays, BPA has been described as a full human AR antagonist and a weak human AR agonist (133).

##### BPA and PPARs

In *in vitro* bioassays, BPA, and the halogenated forms used in flame retardants, such as tetrabromobisphenol A (TBBPA) and tetrachlorobisphenol A (TCBPA), bind to PPARγ (137, 138). The toxicity of those pollutants in the testis and the direct implication of PPARs still need to be demonstrated.

To our knowledge, there are no data showing PPAR involvement in BPA testicular effects. Conversely, PPARγ activation has been associated with BPA-induced adipogenesis (139).

##### BPA and LXRs

LXRs have never been incriminated in the mediation of the toxicological effects of BPA exposure in the testis. However, in mouse 3T3-L1 adipocyte cells, very low BPA doses (1 pM) increase lipid accumulation. This effect is correlated with up-regulation of *SREBP1c* mRNA, a gene that is directly regulated by LXRs, and also of downstream effectors involved in lipid synthesis and homeostasis (140). *In vivo*, rat exposed to BPA during fetal and neonatal life display adipocyte hypertrophy in the perigonadal adipose tissue at 21 dpp and increased SREBP1c expression (141). Moreover, analysis of the hepatic transcriptome of adult male mice exposed to various doses of BPA (5–5000 μg/kg/day in the food) for 28 days showed that low doses of BPA directly increase LXRs and SREBP1c expression and alter fatty acid biosynthesis (142). As LXRs appear to be involved in the regulation of both steroidogenesis and spermatogenesis, it is crucial to further investigate their potential role in the effects on male reproductive functions of BPA exposure during fetal and neonatal life.

##### BPA and SHP

To our knowledge, no data is available on SHP involvement in BPA testicular effects.

## Involvement of Other Receptors and Other Pathways

Based on their reprotoxic effects, most EDCs were thought to have pro-estrogenic or anti-androgenic effects via ERs and ARs (1, 143–145). However, evidences based on transgenic models, genomic analyses, and *in vitro* binding bioassays highlighted the involvement of additional or alternative pathways. This may account for the effects observed at much lower concentrations and for the non-monotonic dose-response often observed with EDCs (146, 147).

Some studies highlighted the involvement of the glucocorticoid and mineralocorticoid receptors (MRs) in the mechanisms of action of phthalates. Indeed, prepubertal exposure to DBP inhibits testosterone production through a glucocorticoid-mediated pathway (148). Moreover, *in utero* exposure to DEHP results in decreased MR mRNA and protein expression in adult interstitial Leydig cells and reduced mRNA expression of MR-regulated genes (149).

The molecular basis of BPA deleterious effects is poorly known, and currently the effects of low BPA doses are the focus of major discussions. Although ERα and ERβ are considered to be the main BPA targets, several other cellular targets have been proposed. Specifically, BPA could be a ligand of estrogen-related receptor gamma (ERRγ) (150) and of G protein-coupled estrogen receptor (GPER) (151). Moreover, low BPA concentrations trigger effects via G-protein coupled receptor 30 (GPR30) (152, 153).

Bisphenol A binds to the orphan nuclear receptor ERRγ at nanomolar concentrations with high specificity (154), and BPA positively regulates the transcriptional activity of human ERRγ. Moreover, these *in vitro* effects of BPA on ERRγ have been observed also *in vivo* during zebrafish development (154). Interestingly, ERRγ has higher affinity for BPA than for DES. The mechanism of action of BPA may be complex and it could also antagonize the binding of an unknown ERRγ ligand, thus maintaining the receptor in a constitutively active form (155).

Both GPR30 and ERRγ are expressed in mouse and human fetal testes. Using sorted cells from mouse 13.5 dpc testes, we observed that ERRγ is mainly expressed in interstitial cells and GPR30 in germ cells at the same level as ERβ (data not shown, Figure 1).

Other endocrine receptors, such as glucocorticoid receptor, thyroid receptor, and aryl hydro-carbon receptor (AhR), are also possible BPA targets, although their *in vivo* relevance is still debated (156).

Diethylstilbestrol has been considered for years as the paradigm of environmental EDCs with estrogen-like activity. Indeed, DES acts through ERs. However, recent studies suggest that LXRα and LXRβ not only are important for testis physiology, but could also exert a protective effect against estrogen-like endocrine disruptors (67).

## New Perspectives

Historically, the concept of EDCs arose from observations in wild fauna. During the last decades, it has become obvious, based on the analysis of the effects of a limited number of compounds, that some chemicals, to which the general population might be exposed, can negatively affect also human health, particularly by targeting the reproductive function and testis development. We suggest that the huge number of different signaling pathways required for gametogenesis and steroidogenesis also might explain/contribute to the high vulnerability of this system to exogenous interferences. One consequence of the wealth of studies on the effects of EDCs on human male reproduction is the growing interest of the general population and of policy makers. This has been translated rapidly into the legal ban of some highly mediatized compounds. While this seemed the proper response at first, in reality, it did not change much the way of consuming, and many less studied or less well-known compounds, which could be as deleterious as those that have been banned, remain in the environment. Even more worrying, “new” molecules for which very few or no data on their potential toxicity exist are put on the market to palliate for the withdrawal of some “old” ones. This increases the diversity and the number of compounds we may be concomitantly exposed to, another risk that is difficult to assess as these compound mixtures change over time. On the other hand, the action of a single EDC may involve directly or indirectly several nuclear receptors, while multiple EDCs may act through a single nuclear receptor. Despite all these difficulties, it is surprising that few studies have investigated the activation of nuclear receptors upon EDC exposure simply by measuring the target gene expression. Indeed, nuclear receptors constitute a large family of transcription factors that regulate developmental and physiological processes by directly controlling gene expression. Moreover, nuclear receptor-mediated transcription is often modulated through tissue-specific coregulators (coactivators or corepressors) (157); however, very few studies have investigated EDC effects on the expression of these coregulators. These issues will have to be addressed in future studies to provide reliable predictions about chemicals suspected to threaten human reproductive health. The formidable explosion of cre-lox-based approaches and transcriptomic analyses has rendered this goal achievable.

Another important point to address is whether it is worth to continue working on specific compounds for which a wealth of scientific data and experimental models are available, even though these compounds are going to be legally banned soon. The answer is unexpectedly yes! For many EDCs, we close to understand the mechanism of reprotoxicity. Indeed, the finding that reprotoxicity is not obligatorily mediated through estrogen- or androgen-disrupting activities allows now focusing on structure-based approach. Additionally, the identification of the mechanisms of action of a given compound is a mandatory step that will allow screening at medium throughput the thousands of remaining pollutants, which may use the same mechanism to target reproductive functions, and help predicting their potential reprotoxic effect. However, this will only be possible if the mechanism underlying their deleterious effect within the reproductive organs, and not only in a reporter cell line, is deciphered. On the other hand, major advances from *in silico*, *in vitro*, and *in vivo* (notably model organisms) studies are now helping to define some unsuspected challenges. For instance, it is now possible to propose that two substances together may activate an unexpected signaling cascade. Such evidences are expected to multiply in the near future and raise a major question: how are we expected to investigate the potential effect of combinations of hundreds of compounds? For instance, if among the 900 existing EDCs, only 10 compounds are sufficiently abundant, this would already lead to billions (9 × 10^22^) of possible different exposure combinations. Is this a lost battle? Possibly not. Indeed scientists, and particularly toxicologists, always aim at performing experiments in specifically defined conditions and by taking into account similar experiments for reasoning. This might be a narrow view and one may wish to test now more realistic exposure conditions with less defined mixtures but that reflect a real life situation. For instance, it has already been proposed to start using sewage sludge and from there move to airborne mixtures, compounds extracted from serum…etc.

In the end, every effort needs to keep in mind human health. From this point of view, the potential reprotoxicity of chemicals is worrying and alarming. The potential transgenerational effect of some substances is an additional issue. Indeed, we may need to determine not only the current exposure but also what our ancestors have been exposed to. This alarming scenery is definitively a factor of stress and stress is an endocrine based/endocrine disrupting situation that affects negatively the reproductive function as well. This is something we all need to consider when communicating to broad audiences. On an optimistic note, we hope that as EDC deleterious effects are under the spotlight, this will help gathering efforts to understand the mechanism of action of some of them. However, this is insufficient and a sustained research effort on the fundamental mechanisms that govern the development of the reproductive system, especially in humans, is obviously needed. Indeed, unraveling the key signaling cascades underlying the development and maintenance of the reproductive function is a complementary approach to identify altered signaling pathways using model compounds. Only, the synergy of both approaches will ensure the efficient prediction of EDC reprotoxicity and preserve the health of the future human generations.

## Conclusion

Despite the wealth of data showing the deleterious effects of phthalates, BPA, and DES on testis development, their mechanisms of action remain poorly understood. In a general way, EDCs can act by modulating many genomic and non-genomic pathways. While non-genomic mechanisms are already seriously investigated, the deregulation of genomic signaling through nuclear receptors has not been fully elucidated yet. Two main reasons could explain this: first, the embryonic lethality of some mutants (e.g., PPARγ) may preclude later investigations; second, the complexity of the system. We believe that it is still essential to identify the mechanism of action of compounds, the harmful effects of which have been already demonstrated in the developing testis. This knowledge might help us predicting the reprotoxic potential of other substances that, alone or in combination with other molecules, use the same mechanism to target reproduction. Such prediction tools are crucially needed to supervise efficiently the replacement of compounds, such as BPA or phthalates, by other molecules that are currently poorly known.

## Conflict of Interest Statement

All the authors have nothing to declare. All the authors or institutions never received any personal payment or service or funding for research studies from any chemical industrial company.
